# Increased Expression of *MERTK* is Associated with a Unique Form of Canine Retinopathy

**DOI:** 10.1371/journal.pone.0114552

**Published:** 2014-12-17

**Authors:** Saija J. Ahonen, Meharji Arumilli, Eija Seppälä, Osmo Hakosalo, Maria K. Kaukonen, András M. Komáromy, Hannes Lohi

**Affiliations:** 1 Department of Veterinary Biosciences and Research Programs Unit, Molecular Neurology, University of Helsinki, Helsinki, Finland; 2 The Folkhälsan Institute of Genetics, Helsinki, Finland; 3 Department of Small Animal Clinical Sciences, College of Veterinary Medicine, Michigan State University, East Lansing, Michigan, United States of America; 4 Department of Clinical Studies, School of Veterinary Medicine, University of Pennsylvania, Philadelphia, Pennsylvania, United States of America; University of Sydney, Australia

## Abstract

Progressive retinal degenerations are among the most common causes of blindness both in human and in dogs. Canine progressive retinal atrophy (PRA) resembles human retinitis pigmentosa (RP) and is typically characterized by a progressive loss of rod photoreceptors followed by a loss of cone function. The disease gradually progress from the loss of night and day vision to a complete blindness. We have recently described a unique form of retinopathy characterized by the multifocal gray/brown discoloration and thinning of the retina in the Swedish Vallhund (SV) breed. We aimed to identify the genetic cause by performing a genome wide association analysis in a cohort of 18 affected and 10 healthy control dogs using Illumina's canine 22k SNP array. We mapped the disease to canine chromosome 17 (p = 7.7×10^−5^) and found a 6.1 Mb shared homozygous region in the affected dogs. A combined analysis of the GWAS and replication data with additional 60 dogs confirmed the association (p = 4.3×10^−8^, OR = 11.2 for homozygosity). A targeted resequencing of the entire associated region in four cases and four controls with opposite risk haplotypes identified several variants in the coding region of functional candidate genes, such as a known retinopathy gene, *MERTK*. However, none of the identified coding variants followed a compelling case- or breed-specific segregation pattern. The expression analyses of four candidate genes in the region, *MERTK*, *NPHP1*, *ANAPC1* and *KRCC1*, revealed specific upregulation of *MERTK* in the retina of the affected dogs. Collectively, these results indicate that the retinopathy is associated with overexpression of *MERTK*, however further investigation is needed to discover the regulatory mutation for the better understanding of the disease pathogenesis. Our study establishes a novel gain-of-function model for the *MERTK* biology and provides a therapy model for retinopathy *MERTK* inhibitors. Meanwhile, a marker-based genetic counseling can be developed to revise breeding programs.

## Introduction

Dogs suffer from hundreds of hereditary disorders according to the Online Mendelian Inheritance in Animal database (OMIA, http://omia.angis.org.au/home/) and many of them represent clinically and physiologically relevant models for human conditions. Examples include several retinal conditions, such as canine multifocal retinopathies (cmr) [1]–[2] and Leber congenital amaurosis (canine LCA) [3]. Progressive retinal degenerations form a heterogeneous group of disorders that affect different retinal cells such as photoreceptors or retinal pigment epithelium (RPE), resulting in the impairment or complete loss of vision (RetNet; http://www.sph.uth.tmc.edu/Retnet/). Retinitis pigmentosa (RP) is one of the most common incurable blindness worldwide [4]. In RP, the degenerative process typically starts from rod photoreceptors and expands to cone cells leading to a progressive loss of both night- and day light vision before complete blindness [5].

Canine progressive retinal degenerations resemble human RP and are commonly referred as progressive retinal atrophies (PRA). PRA affects many breeds with remarkable variation in the etiology, progression and onset. Careful characterization of these conditions across breeds is not only important for the health of the dogs but could also provide valuable information about the genetics, retinal biology, molecular pathogenesis of RPs and possible environmental factors complementing existing human studies. Furthermore, gene discoveries would establish large animal models for retinal gene therapies [6]–[7]. Today, over dozen PRA genes have been described in dogs [1], [3], [8]–[23], and many remain still to be found.

We have recently characterized a unique type of retinal degeneration in the Swedish Vallhund (SV) breed [24]. (**S1 Figure**). The phenotype of this disease differs from most known forms of PRA with a multifocal rather than diffuse degeneration of the retina. Furthermore, age of onset and rate of progression vary considerably even in the littermates. Clinical signs progress in three stages ranging from diffuse multifocal red/brown discoloration of the tapetal fundus without associated visual deficits (Stage 1), to geographic retinal thinning/degeneration with mild to moderate signs of night-blindness (Stage 2), to more diffuse retinal thinning/degeneration affecting most of the tapetal fundus and associated with night-vision loss and severely impaired day-vision (Stage 3) [24]. This disease affects both the RPE and rod and cone photoreceptors with an excessive accumulation of autofluorescent material within the RPE [24]. Since the known canine PRA genes did not associate with the disease [24], we embarked a study here to identify the genetic cause.

## Materials and Methods

### Study cohort

Blood samples from SVs across various countries were collected to the canine DNA bank at the University of Helsinki, Finland with owner's consent and under the permission of animal ethical committee of County Administrative Board of Southern Finland (ESAVI/6054/04.10.03/2012). Altogether 436 samples were collected, including 93 cases and 76 controls. All affected dogs were examined by certified veterinary ophthalmologists at least once in Finland, Sweden or USA and diagnosed with SV retinopathy. All the control dogs used in the genome-wide association analysis were over 7 years of age at the time of eye examination by veterinary ophthalmologists and none of them were diagnosed with any retinal abnormalities. Genomic DNA was extracted from EDTA blood samples using Chemagic Magnetic Separation Module I (MSM I) (Chemagen Biopolymer-Technologie AG, Baeswieler, Germany) according to the manufacturer's instructions.

Retinal samples from four affected SVs and a PRA-free Australian Cattle Dog and a Belgian Shepherd became available due to euthaniziation for unrelated causes, and were collected post mortem with owners' consents. RNA was extracted using RNeasy Mini Kit (Qiagen) according to manufacturer's instructions.

A pedigree (**S2 Figure**) modified from related manuscript, Cooper et al. [24] (**S1 Figure**) was constructed around the affected dogs using Genopro software and the genealogical data available in public canine registries such as the Finnish Kennel Club's Koiranet, the Swedish Kennel Club's Hunddata databases or as informed by the owners.

### Genome wide association study

To map the retinopathy locus in SVs a genome-wide association mapping was performed with 18 cases and 10 controls using Illumina Canine SNP20 BeadChip array (San Diego, CA, USA). Genotyping was performed at the FIMM Technology Center. The genotyping data was analyzed using PLINK 1.07 analysis software [25]. A total of 22,362 markers were initially included for the analysis. No individual were removed for low genotyping success of 95%. Missingness test of 95% removed 87 SNPs and the average genotyping rate per individual remained at 99.9%. A total of 8,078 SNPs had minor allele frequency of less than 5% and were removed. None of the SNPs deviated from Hardy-Weinberg equilibrium based of HWE test of P< = 0.0001. After frequency and genotyping pruning, 13,699 SNPs remained in the analysis.

To compare the affected dogs and healthy control dogs an allelic case-control association test was performed and significance values from this analysis were used to generate a whole-genome association plot using R-program [26]. Identity-by-state (IBS) clustering and CMH meta-analysis (PLINK) were used to adjust for population stratification. Genome-wide corrected empirical p-values were determined applying 50,000 permutations to the data. Besided PLINK the data was analyzed using R-implemented GenABEL software [27] (data not shown).

### Replication study

A replication study for the best associated SNPs at CFA17 (BICF2G630207991) was performed in additional 34 cases and 26 controls. We performed a standard PCR, including 1.2 U Biotools DNA Polymerase (Biotools, Madrid, Spain), 1.5 mM MgCl2 (Biotools, Madrid, Spain), 200 µM dNTPs (Finnzymes, Espoo, Finland), 1× Biotools PCR buffer (Biotools, Madrid, Spain), 0.83 µM forward GCTGCTTCCTTTTTGCTCAT and reverse GGTGCTACGTTTGACAGCAA primers (Sigma Aldrich, St. Louis, USA) and 10 ng template genomic DNA. Reaction mixtures were subjected to a thermal cycling program of 95°C for 10 min, 35 cycles of 95°C for 30 s, 30 s 58°C, 72°C for 60 s and a final elongation stage of 72°C for 10 min. ExoSap (USB Corporation, Ohio, USA) purified fragments were sequenced at the FIMM Technology Center using ABI 3730xl DNA analyzer (Applied Biosystems, Foster City, California, USA). Sequence analysis was performed using Variant Reporter software (Applied Biosystems, Foster City, California, USA).

### Targeted capture and re-sequencing

**Figure 1:**
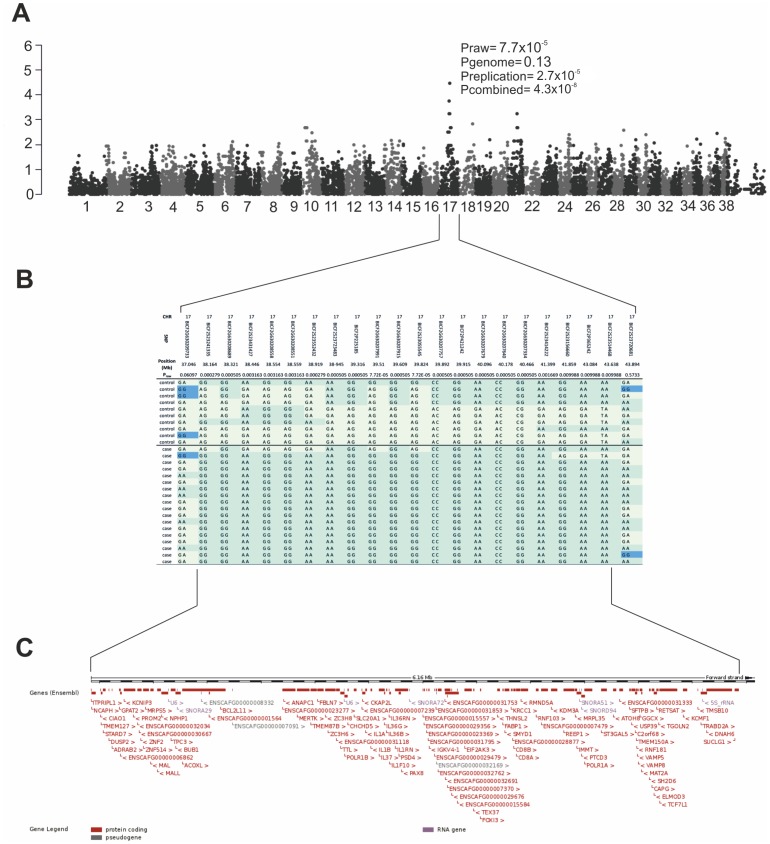
Results of the genome-wide association study. Genome-wide association analysis identifies the retinopathy locus in the SV breed. **A**) Manhattan plot with tentative association on CFA17 (p_raw_ = 7.7×10^−5^), replication analysis supported the association (p_repl_ = 2.7×10^−5^) and was confirmed by combined analysis (p = 4.3×10^−8^). CFA39 represent the X chromosome. **B**) A close-up of the associated region on CFA17, which spans from 38.16 Mb to 43.64 Mb. **C**) The associated region harbors over hundred genes, including a known PRA gene, *MERTK*.

The associated region of 6.1 Mb on CFA17 (37.53–43.64 Mb, CanFam2.0) was resequenced in four affected SVs, four healthy SVs and 16 dogs from two other breeds, Dandie Dinmont Terrier and Staffordshire Bull Terrier. The SV samples were selected based on the opposite haplotypes in cases and controls (**Fig. 1B**). The region was captured using custom designed probes (according to the build 2.1 of the canine genome reference sequence) and Roche Nimblegen solution based capture method followed by paired-end sequencing using the Illumina HiSeq2000.

The data analysis included quality control, alignment, variant calling and annotation of the variants. Quality control was performed using FASTX toolkit (http://hannonlab.cshl.edu/fastx_toolkit/index.html) to remove the low quality bases called by the sequencing machine. Base call accuracy of 99% i.e. bases with Phred scores <Q20, were trimmed to reduce false positives during variant calling. The quality passed paired-end reads were aligned to the build 3.1 of the canine genome reference sequence with Burrows-Wheeler (BWA) aligner tool with default parameters [28]. After mapping, the reads that mapped to the targeted region were extracted followed by the removal of potential PCR duplicate reads using Samtools 0.1.18 [29]. Local realignment around potential indel sites and base quality scores recalibration were implemented using GATK [30] and fix mate-pair information using Samtools [29] to improve the quality of the sequence data before variant calling. The sequence alignments were then directed to the variant calling programs GATK [30] and Samtools [29] to identify the SNPs and short indels present within the samples. Pindel program was further used to identify indels and structural variants [31]. Finally, the identified variants were annotated against NCBI and UCSC databases to find out the variants present in the coding and non-coding regions. The annotation was performed using SnpEff [32] and in-house custom R-scripts. The dbSNP131 database was utilized to identify known polymorphic variants. To identify the case-specific variant, the data was filtered against the controls and 16 other dogs from two breeds under a recessive model using our in-house R-scripts. To study the CNVs and repeat elements (SINE, LINE), a heatmap analysis was performed by comparing normalized read depths in each position between cases and controls.

### Validation of candidate causative variants

The tentative disease causing variants identified by resequencing, were genotyped in a large sample cohort of SVs (**Table 1**) and from eight unaffected dogs from two other breeds for the breed specificity. The identified variants were categorized based on type and position: non-synonymous first, followed by variants in the non-coding RNAs, UTR regions, and conserved intergenic and intronic regions, respectively (**Table 1**). The primers for the validated markers are available upon request. The association of each variant with the disease was calculated by PLINK [25].

**Table 1:** Markers selected for further studies based on the targeted resequencing data.

CODING																
Gene	Type	Position †	Ref	Alt	Protein	Polyphen2/SIFT prediction	No. Case	No. Control	P	OR	CHISQ	HMM based conservation score	A1	F_A	F_U	A2
*NPHP1*	SNV	35128247	A	G	p.H86R	probably damaging	51	33	5.2×10^−6^	5.7	20.8	0.4	G	0.9	0.6	A
*ANAPC1*	SNV	36279583	G	A	p.V734I	benign	51	33	0.09	1.7	2.8	0.3	A	0.7	0.6	G
*MERTK*	SNV	36405419	G	C	p.A369P	benign	51	33	2.8×10^−6^	6.2	21.9	1.0	C	0.9	0.6	G
*KRCC1*	SNV	38321727	T	G	p.L97R	probably damaging	51	33	6.0×10^−4^	4.8	11.9	0.5	G	0.9	0.7	T
NON-CODING																
*BCL2L11*	INDEL	35627062–35627089	ACACTTTCAGTTCTTTTGGATATCTAT	-			23	22	0.01	3.2	6.7	0.01	del/del	0.8	0.5	ref
*MERTK*	SNV	36348610	A	G			30	33	0.04	2.5	4.1	-	G	0.9	0.7	A
*MERTK*	INDEL	36348691	-	TCTG			23	23	0.001	4.5	10.6	-	ins/ins	0.8	0.5	ref
*MERTK*	SNV	36410244	C	A			23	23	0.002	3.9	9.1	0.02	A	0.8	0.5	C
*MERTK*	SNV	36410251	T	C			23	23	0.004	3.8	8.2	0.02	C	0.8	0.5	T
*SLC20A1*	SNV	36872452	C	T			22	20	0.003	3.8	8.9	0.007	T	0.8	0.5	C
*PSD4*	SNV	37267152	A	T			21	18	0.05	2.8	3.8	0.2	T	0.8	0.6	A
*ncRNA*	SNV	37464374	A	G			10	6	0.0003	5.8	13.3	0.007	A	0.7	0.3	G
*ENSCAFG00000028877*	SNV	38310169	A	G			19	19	0.009	5.4	6.7	0.05	A	0.9	0.7	G
*ENSCAFG00000028877*	SNV	38310174	G	A			36	31	0.002	6.1	9.4	0.06	G	1.0	0.8	A
*ENSCAFG00000028877*	SNV	38310216	G	A			20	18	0.02	4.0	5.8	0.005	G	0.9	0.6	A
*ENSCAFG00000028877*	SNV	38310235	A	T			39	27	0.006	3.7	7.5	0.2	A	0.9	0.7	T
*ENSCAFG00000028877*	SNV	38310249	T	C			39	32	NA	NA	NA	0.2	C	1	1	T
*RNF103*	SNV	38568101	A	G			20	20	NA	NA	NA	0.09	A	1	1	G
*RNF103*	SNV	38569756	A	C			21	21	0.1	2.1	2.6	0.007	C	0.7	0.6	A
*RNF103*	SNV	38570219	G	A			20	20	0.1	2.2	2.7	0.3	A	0.8	0.6	G
*KDM3A*	INDEL	38601421	-	GTGGA			23	22	0.2	1.7	1.9	0.04	ins/ins	0.7	0.5	ref
*KDM3A*	SNV	38753172	G	A			28	24	0.2	1.7	1.6	0.05	A	0.7	0.5	G
*KDM3A*	SNV	38753853	G	A			23	22	0.2	1.7	1.6	0.02	A	0.7	0.5	G
*KDM3A*	SNV	38753998	A	G			23	22	0.2	1.7	1.6	0.1	G	0.7	0.5	A
Gene	Type	Position †	Ref	Alt	Protein	Polyphen2/SIFT prediction	No. Case	No. Control	P	OR	CHISQ	HMM based conservation score	A1	F_A	F_U	A2
-	INDEL	38785175	AGGT	-			15	16	0.6	0.8	0.2	0.01	ref	0.6	0.6	del/del
*REEP1*	INDEL	38906987–38906990	TACT	-			23	23	0.3	1.6	1.2	0.9	del/del	0.7	0.6	ref
*POLR1A*	SNV	39044115	C	T			26	24	0.4	1.4	0.8	0.1	T	0.6	0.6	C
*POLR1A*	SNV	39044233	G	A			26	22	0.3	1.6	1.1	0.2	A	0.7	0.6	G
*POLR1A*	INDEL	39048757	-	A			23	23	0.3	1.6	1.2	0	ins/ins	0.7	0.6	ref
*SH2D6*	SNV	39603031	A	T			23	21	0.6	1.7	0.3	0.2	T	1	0.9	A
*SH2D6*	SNV	39603158	C	A			31	23	0.2	4.3	2.8	0	A	1	0.9	C
*SH2D6*	SNV	39613315	G	A			22	21	0.6	1.4	0.2	0.009	A	0.9	0.9	G
*SH2D6*	SNV	39613514	T	C			20	19	0.1	4.6	2.1	0.007	C	1	0.9	T
*TGOLN2*	SNV	39685675	C	T			23	22	0.3	2.3	1.3	0.03	T	0.9	0.8	C

+ The position is based on canine reference sequence CanFam3.1.

The best association was found in the *MERTK* gene.

### Expression analyses

Retinal RNA samples were studied to screen *MERTK* transcript for mutations and to quantitate transcript levels of four retinal candidate genes, *MERTK*, *NPHP1*, *ANAPC1* and *KRCC1* between the affected (n = 4) and unaffected (n = 2) dogs. The *MERTK* mRNA sequence (XM_005630437.1) was amplified using Biotools (Biotools, Madrid, Spain)) polymerase and PCR protocol described in the replication section with annealing temperature of 60°C. Amplicons were Sanger sequenced for mutations.

To quantitate transcript levels, a real time PCR was performed using Applied Biosystems'7500 Fast Real-Time PCR machine and Universal SYBR Green Master (Roche). RT-PCR was carried out on equal amounts of retinal RNA in each sample by using the High Capasity RNA-to-cDNA kit (Applied Biosystems). RT-PCR was carried out in 0.25 µM of forward and reverse primes in a total reaction volume of 20 µl. The primers used for mRNA amplification and real time PCR are available upon request. A housekeeping gene, GAPDH was used as a normalization control, and triplicate samples were used for all reactions. The efficiency of the reaction was calculated from a seven-point dilution series. No significant differences were detected in the efficiencies between the housekeeping and target reactions, and the comparative ΔΔCt-method could be used to determine relative expression differences. Statistical significance of the expression differences was calculated by using the Student's t-test on normalized mean cycle threshold (Ct) -values. PASW Statistics 18 software SPSS (IBM) was used to perform the statistical tests.

## Results

We have recently identified a novel type of retinal degeneration in SVs with a likely recessive mode of inheritance [24] and aimed to map the disease locus here by expanding the SV study cohort across different countries in Europe and the US. We performed a GWAS in a cohort of 18 cases and 10 controls using Illumina's canine 22k SNP array. Genotyping data was analyzed by a linear regression (PLINK) and mixed model approaches (GenAbel, data not shown). No significant population structure was identified by genome wide IBS clustering (λ = 1.1). We identified a tentative locus on CFA17 (p_raw_ = 7.7×10^−5^, p_genome_ = 0.13) with the most highly associated SNP, BICF2G630207991 (**Fig. 1A**).

To confirm the association, we genotyped the best associated SNP on CFA17 in additional 34 cases and 26 controls (p_repl_ = 2.7×10^−5^). The combined analysis (GWAS and replication) supported the association (p_com_ = 4.3×10^−8^) (**Fig. 1A**).

According to the combined analysis, 82% (41/50) of the cases and 30.5% (11/36) of controls were homozygous for the risk allele at CFA17 given the (OR = 11.2). Eleven unaffected dogs that were homozygous for the risk allele were all eye examined healthy after 6 years of age, however, it is possible that the dogs become affected later as the age of onset and disease progression varies even in the littermates [24]. Reduced penetrance of the disease may be linked to the large phenotypic variation or possible genetic or environmental modifiers [24].

### Candidate gene analysis

The associated region has a homozygous risk haplotype in the cases (17/18), spanning a 6.1 Mb region from 37.5 Mb to 43.60 Mb (**Fig. 1B**). One of the cases clearly carries a different haplotype from the rest of the cases and could be a phenocopies due to other reasons (**Fig. 1B**). The region contains 102 genes of which *anaphase promoting complex subunit 1* (*ANAPC1*) [33], *c-mer proto-oncogene tyrosine kinase* (*MERTK*) [34]–[41] and *nephronophthisis 1* (*NPHP1*) [42]–[43] have been previously associated with retinal disease or retinal function (**Fig. 1C**). These genes were selected for exonic mutation screening, revealing three non-synonymous coding variants p.A369P (g.36405419) in *MERTK*, p.H86R (g.3512824) in *NPHP1* and p.V734I (g.36279583) in the *ANAPC1* (**Table 1**).

The pathogenicity of the coding variants was predicted based on the bioinformatics prediction softwares Polyphen2.0 [44] and SIFT [45], which evaluate the evolutionary conservation of the residues across species. The *MERTK* p.A369P and the *ANAPC1* p.V734I variants were predicted to be benign while *NPHP1* variant as likely pathogenic. However, when we genotyped all three coding variants in 33 eye examined unaffected SVs (**Table 1**) and eight dogs from two other unaffected breeds, Whippet and Finnish Lapphund, we found all of them in unaffected SVs and in the two other breed with a moderate frequency, suggesting them as polymorphisms rather than causative.

### Targeted resequencing

To identify the causative mutation, we performed a targeted resequencing of the entire 6.1 Mb associated locus in eight SVs and 16 other dogs from Dandie Dinmont Terrier and Staffordshire Bull Terrier breeds. We reached an average 98.8% sequence coverage across the locus in each dog and found altogether 86,160 single nucleotide variants (SNVs) and 2,294 indels (**S1 Table**). After filtering the variants under the recessive model altogether 408 SNVs and 70 indels were shared between the SV cases (**S1 Table**). The canine reference sequence (CanFam3.1) was used to annotate the identified variants. Although 102 genes have been annotated in the capture region, targeted resequencing revealed only one additional new case specific coding variant, p.L97R, in the *lysine-rich coiled-coil 1* (*KRCC1*) gene (XP_005630523.1). Further analysis of this variant in 51 additional affected and 33 unaffected SVs and in two other unaffected breeds, Whippet and Finnish Lapphund, did not support segregation and indicated that is found in other non-affected breeds (**Table 1**).

Since the identified coding variants did not segregate with the disease, we next selected 30 non-coding variants across conserved (UCSC conservation scores) [46] regions of the top candidate genes for further analyses in additional samples (**Table 1**). However, none of these variants segregated either with the disease.

Possible presence of case-specific CNVs and repeats such as, SINEs and LINEs were studied by a read depth-based heatmap analysis between the cases and controls. No case-specific differences were found in the read depths across the associated region (data not shown).

Collectively, these genomic analyses did not reveal a causative mutation but identify the strongest association with the SNP (BICF2G630207991) in the intron of the *MERTK* gene. This marker was therefore further tested in 400 SVs from our DNA bank revealing a very high carrier frequency (59.9%) and a significant risk for homozygosity (p = 6.3×10^−27^, OR = 18.5 with 95%CI = 10.3–33.2).

### *MERTK* transcript analysis

Because our genomic analyses did not reveal the causative mutation, we decided to sequence the *MERTK* mRNA for possible abnormal splicing or other events from the retinal samples, including the neuro-retina and RPE of two affected SVs and an unaffected dog available. Sequencing revealed the p.A369P variant found in the genomic analyses in the affected dogs but did not reveal any additional variants or abnormal splicing events.

### Expression analysis of the retinal candidate genes

**Figure 2:**
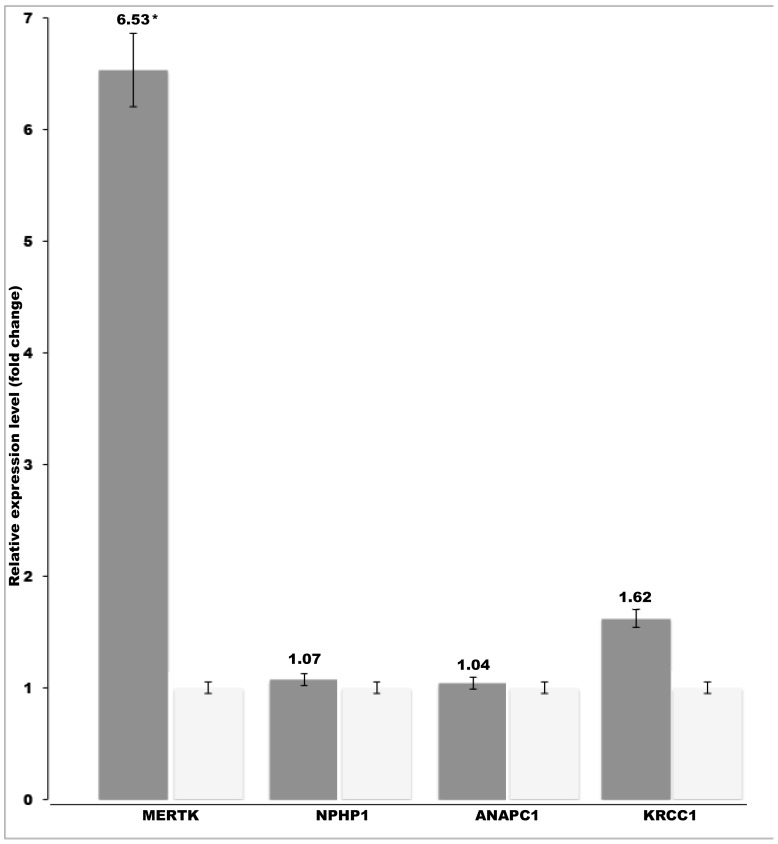
Retinal upregulation of *MERTK* in the affected SVs. The retinal mRNA levels of *MERTK*, *NPHP1*, *ANAPC1* and *KRCC1* genes were compared between affected (n = 4) and unaffected dogs (n = 2). A specific overexpression of *MERTK* was found in the affected SVs. The relative mRNA expression levels are represented as a fold change. Error bars denote the standard error of normalized Ct-values. *p≤0.0001 (two-tailed t-test p-value).

We obtained retinal tissue samples from four retinopathy affected SVs and two control dogs and used them to quantitate transcript levels of the four retinal candidate genes, *MERTK, NPHP1, ANAPC1* and *KRCC1* in the critical region. Real-time PCR revealed a 6.5-fold upregulation of the *MERTK* gene in the affected dogs while no difference was found in the *NPHP1, ANAPC1* or *KRCC1* genes (**Fig. 2**). This result suggests that a regulatory mutation outside the coding region results in the overexpression of *MERTK*, which then leads to retinopathy in the affected dogs.

## Discussion

We have previously identified a unique form of PRA in the SV breed and show here genetic and functional evidence that the disease is associated with a known human RP gene, *MERTK*. The SV breed is small worldwide, which is reflected with extensive linkage disequilibrium of the associated region. The region harbors over 100 genes, including some that have been associated with retinal function or retinal degeneration in human and rodent models, such as *ANAPC1*, *MERTK* and *NPHP1*. Although we found several coding and regulatory variants in these retinal candidate genes, none of them followed appropriate segregation pattern and were present in other unaffected breeds, excluding them as candidates for the disease. However, while the causative variant remains to be found, positional and functional evidence support the role of *MERTK* in the SV retinopathy.

First, our genetic data identified the best association within the *MERTK* gene, and this was 100 times stronger than in any other regions of the gene-rich locus. This result strongly suggests that the cause of the disease lies in the identified region within or nearby *MERTK*. Second, from the three retinal genes in the critical region, *MERTK* appears as the most likely candidate based on its function and clinical significance in the retinal degeneration in human and rat [35], [39], [41], [47]. The MERTK protein is a member of the TAM receptor tyrosine kinase and is expressed in the retinal pigment epithelium (RPE) plasma membrane [48]. It participates in apoptotic cell clearance and cytoskeleton regulation with anti-inflammatory properties [49]. Human recessive *MERTK* mutations have been associated with several phenotypes including RP, rod cone dystrophy and early onset, childhood blindness [34], [36]–[38], [47], [50]–[57]. Progressive loss of photoreceptor cells in *MERTK*-deficient Royal College of Surgeon (RCS) rats was suggested to result from the lack of the cooperation between the photoreceptor and the RPE cells [35].

The analysis of the *MERTK* transcript isolated from the affected retinas indicated an intact transcript sequence without additional coding sequence or splicing abnormalities. However, an unexpected 6.5-fold up-regulation of the *MERTK* transcript was found in the affected retina. This was specific to *MERTK*, since no changes were found in the transcript levels of the other three candidate genes, *NPHP1*, *ANAPC1* and *KRCC1* in the same retinal tissues (**Fig. 2**).

Potential explanations of the increased transcription of *MERTK* might include i) the presence of a regulatory mutation in the *MERTK* gene, ii) a massive infiltration of *MERTK* positive inflammatory cells into the RPE [49], iii) normal variation in the *MERTK* expression due to circadian rhythm of the RPE phagocytosis peaking at the daily onset of light [58] or iv) the presence of a regulatory variation outside *MERTK* affecting its ligands or *MERTK*-induced cellular physiology [59]. The hypotheses ii and iii appear unlikely, since our routine histopathology was not indicative of invasion of inflammatory cells in the affected retina [24], and the retinal samples for the transcript quantification were harvested several hours after the phagocytosis peak when *MERTK* should not be active and elevated. Our genetic data points to a cause within *MERTK* not outside. The presence of a *MERTK*-specific regulatory mutation is more likely and our future efforts will focus on the *MERTK* gene and surrounding regions, including the promoter region, upstream enhancers and possible UTR miRNA binding sites. Overexpression of MERTK has been associated with the variants in the 3UTR miRNA binding sites [60].

Previous *MERTK-*related retinal degenerations have been associated with recessive loss-of-function mutations [34], [36]–[38], [47], [50]–[57]. The question how overexpression of *MERTK* may lead to the multifocal retinopathy in the affected SVs remains unknown, although several possible hypotheses can be speculated based on the diversity of the *MERTK* functions in various models.

First, *MERTK* can activate several intracellular canonical signaling pathways, including phosphoinositide 3 kinase, PI3K/AKT, pathway including phospholipase C, ERK1/2, Ras, and MAP kinase and JAK/STAT pathway [61], which could lead to increased or abnormal apoptosis in the local regions of the RPE.

Second, overexpression of *MERTK* has been shown to induce efferocytosis in cancer cell lines [59]. Similarly, overactive *MERTK* in the RPE could increase the efferocytosis of the POS, lead to an accumulation of photoreceptor debris in the RPE and subsequent degeneration of photoreceptors in the affected dogs. Human *MERTK* patients show deposit of autofluorescence consisting of photoreceptor outer segment (POS) membranes and their by-products, including accumulating lipofuscin in the outer retina [41], [51]. Similarly in RCS rats, an abnormal accumulation of outer segment debris between photoreceptor outer segment layer and the RPE occurs, prior to photoreceptor cell death [62]–[64]. In the RCS rats, RPE cells fail to engulf POS, which causes the accumulation of POS debris in the subretinal space [65]. The SV retinopathy affects both the RPE and rod and cone photoreceptors with an excessive accumulation of autofluorescent lipofuscin-like material within the RPE [24].

Third, the *MERTK* receptor has a soluble form, sMer [66] with an antagonistic role to full-length MERTK. sMer can bind to Gas6 and inhibit Gas6-mediated *MERTK* activation [66], which in turn could result in the impaired phagocytosis and retinopathy. sMer is posttranslationally generated from the MERTK receptor by the ADAM17 cleavage [66] and its expression patterns in the affected retina and RPE should be studied at the protein level.

Fourth, *MERTK* overexpression has been shown to result in an altered localization of the *MERTK* protein in the nuclear compartment [67]. In the RPE, this model could lead to a net loss-of-function effect of the *MERTK* activity as seen in the recessive RP cases.

Finally, *MERTK* is expressed in macrophages that participate in the phagocytosis [68]. Constitutive overexpression of *MERTK* in the macrophages could result in the enhanced local efferocytosis in the RPE, which in turn, would further promote MERTK activity and undesired apoptosis and loss of photoreceptors.

Upon confirmation of the *MERTK* overexpression with subsequent downstream mechanisms, existing MERTK inhibitors [69] may provide a therapeutic option for the retinopathy dogs. *MERTK* overexpression is characteristic to several cancers and inhibition in those models has been found efficient [69]–[70]. A clinical trial with *MERTK* inhibitors remains as an exciting possibility not only to treat canine patients but also to better understand the related disease mechanisms and *MERTK* biology in the eye.

The uniqueness of the SV retinopathy is based on the retinal disease phenotype, the variation in age of onset and in the rate of disease progression. This suggests that genetic and/or environmental disease modifiers likely contribute to the disease phenotype. Our GWAS data with a modest samples size revealed a single locus with a possible reduced penetrance. The vast majority of the affected dogs are homozygous for the risk haplotype, however, the phenotypic variability in SVs may be related to the particular type of regulatory mutation causing the upregulation of *MERTK* in combination with possible environmental factors.

Three additional coding variants were found in other genes. A predicted pathological variant, p.H86R, was identified in the *NPHP1* gene, which encodes for *nephronophthisis 1*. *NPHP1* is widely expressed in many tissues and localized to the photoreceptor-connecting cilia at the junction of the inner segment and outer segment [71]. The gene has been implicated in an autosomal recessive, juvenile nephronophthisis 1 [72] with infrequent retinal degeneration [73]. *Nphp1*-deficient mice present an early-onset rapidly progressing degeneration of the outer and inner segments and nuclei, losing the photoreceptors within the first 8 months of life [74]. Although the p.H86R variant was predicted to be pathological, its homozygous presence in other unaffected SVs and breeds exclude its causative role.

The third coding variant (p.V734I) was identified in the *ANAPC1* gene. This gene has been implicated in the normal eye development in Drosophila. The *shattered* (*shtd*) mutation in the fly leads to a failure in G1 arrest during the mitosis, causing a defective arrangement of photoreceptor cells and other developmental problems in the eye [75]. Again the identified variant in our SVs was not case- or breed-specific ruling it out as the cause of the disease.

The fourth non-synonymous coding variant in the associated region, p.L97R, was found in the *KRCC1* gene, which encodes a lysine-rich coiled-coin protein 1, with an unknown function. However, the *KRCC1* variant was also present in other unaffected dogs and did not segregate with the disease.

Similarly, a large number of potential regulatory variants were found in the conserved regions across the associated region but validation experiments for 30 of them did not support an appropriate segregation pattern. We may have missed the causative mutation in *MERTK* due to technical reasons despite high quality resequencing data. Targeted capture is not efficient in repetitive regions, which are often lost already at the target design. Another challenge relates to possible larger structural variants such as CNVs, which are not easy to pinpoint in the resequencing data. It is possible that some coding variants have been missed in our analysis, however, the annotations around *MERTK* locus were identical in both species and it is unlikely that coding variants were missed in the critical region. We will consider resequencing of the whole genomes of SVs to avoid capture-related obstacles.

In conclusion, we have mapped the cause of retinopathy in SVs and show the involvement of the up-regulated *MERTK* gene in the affected dogs. Our study establishes a novel gain-of-function model for the *MERTK* physiology in the retina. Future studies will include the search for the regulatory mutation and study of overexpression-related disease mechanisms with a possibility for a therapeutic option with MERTK inhibitors. Meanwhile, a genetic marker test can be developed for breeding purposes to help the future SV population to reduce the high-risk allele frequency in the breed.

## Supporting Information

S1 Figure **A pedigree from a related manuscript, Cooper et al., indicates clinically studied dogs** [24] **.**
(TIF)

S2 Figure **Pedigree indicates the dogs that were used in the GWAS study (marked yellow). Disease segregation suggests an autosomal recessive mode of inheritance.**
(TIF)

S1 Table Summary of the targeted resequencing data. The 6.1 Mb associated region was captured and resequenced in four cases and four control SVs with opposite risk haplotypes to identify the causative mutation.
(XLSX)
