# Supplementary figures and images for: Increased Expression of MERTK is Associated with a Unique Form of Canine Retinopathy
Source: PLoS One. 2014 Dec 17;9(12):e114552. doi: 10.1371/journal.pone.0114552 (PMC4269413; doi:10.1371/journal.pone.0114552)

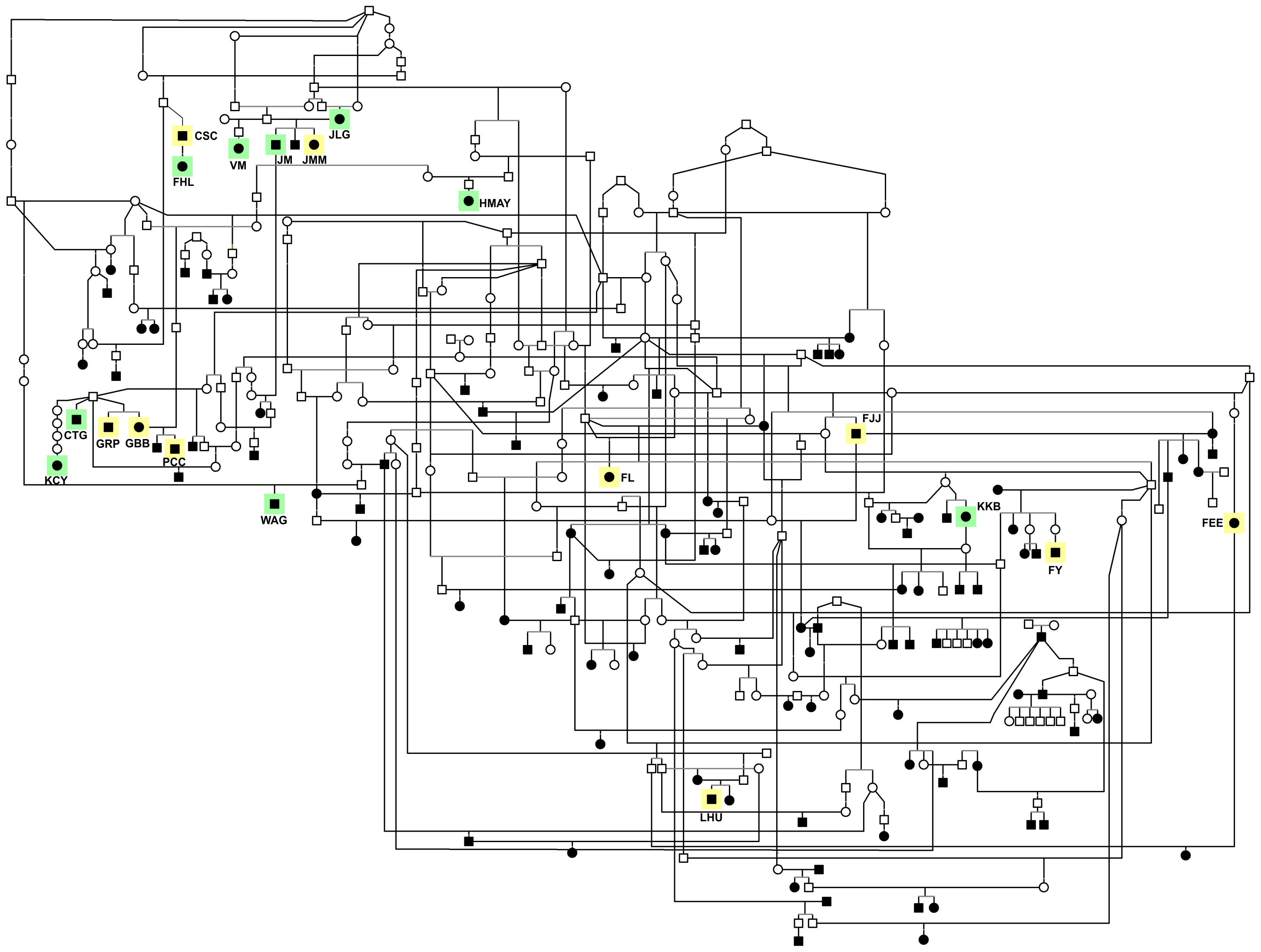

Supplement: S1 Figure — A pedigree from a related manuscript, Cooper et al., indicates clinically studied dogs [24] . (TIF) [file pone.0114552.s001.tif]

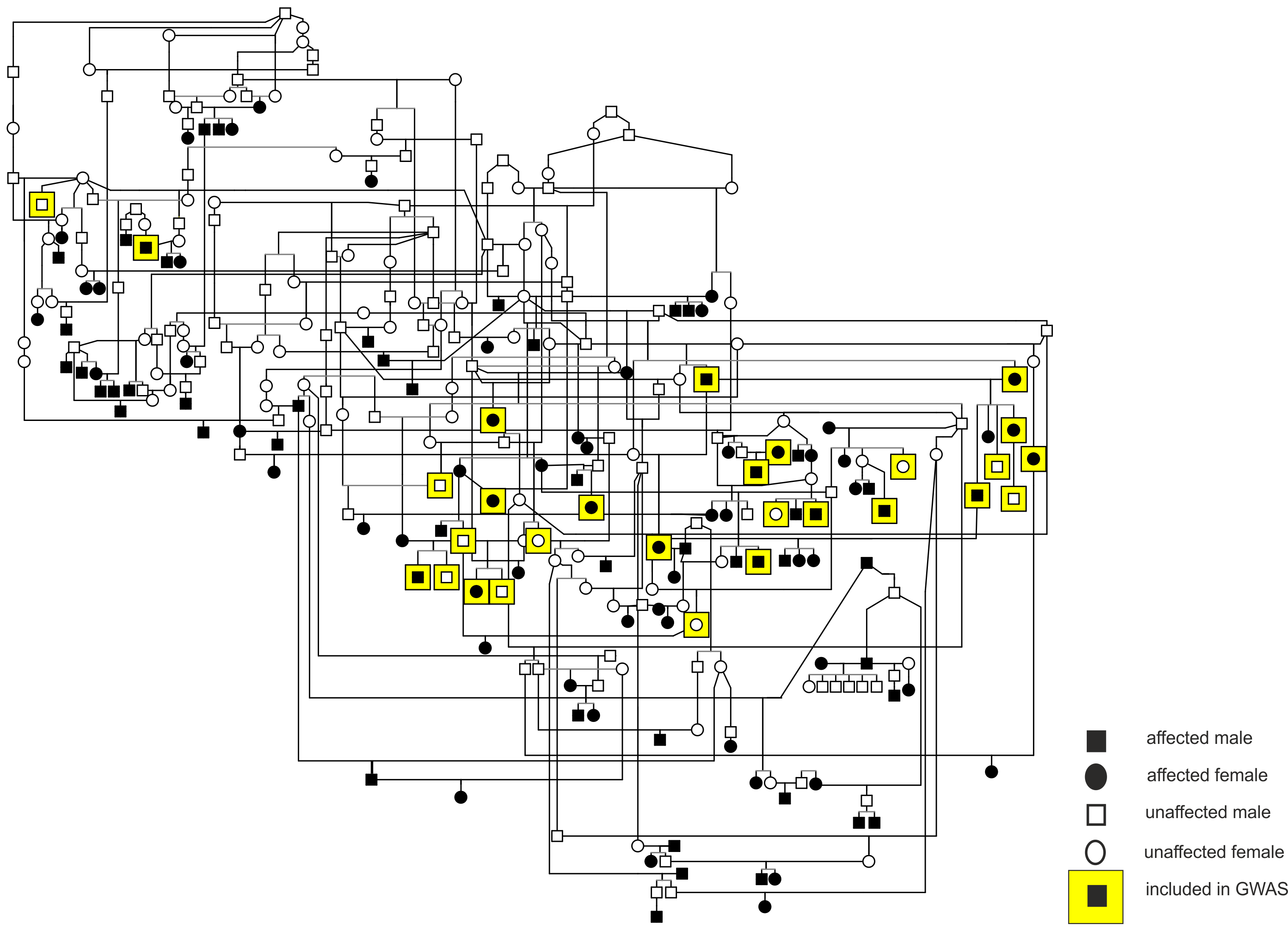

Supplement: S2 Figure — Pedigree indicates the dogs that were used in the GWAS study (marked yellow). Disease segregation suggests an autosomal recessive mode of inheritance. (TIF) [file pone.0114552.s002.tif]
